# Supplementary material for: Behavioral evidence for memory replay of video episodes in the macaque
Source: eLife. 2020 Apr 20;9:e54519. doi: 10.7554/eLife.54519 (PMC7234809; doi:10.7554/eLife.54519)
Supplement: Supplementary file 1. [file elife-54519-supp1.docx]

Monkey data:

**Data description**

| **Variable Name** | **Value Explanation** |
| --- | --- |
| Play_condition | ‘0’=normal; ‘1’=reverse |
| Temporal_distance | 1 to 25 correspond to 25 TD levels |
| boundary | ‘0’=within; ‘1’=across |
| response_correct | ‘1’=correct; ‘0’=incorrect |
| mouse.time | RT |
| delay1 | The delay time of correct answer (ms) |
| delay2 | The delay time of the other frame (ms) |
| Fresh/Repeat | 1 to 5 correspond to 5 exposure levels |
| Nonprimate/Primate | ‘0’=primate; ‘1’=non-primate |
| Monkey | 1 to 6 correspond to Jupiter, Mars, Saturn, Mercury, Uranus, Neptune. |
| chosen_frame_location | The frame number of which the monkey has chosen in each trial. |
| Target_frame_location | The frame number of correct answer in each trial. |
| hist_dist, hog, surf | hist_dist, hog and surf are three index of perceptual similarity, which corresponds to RGB-histogram, HOG, SURF in Figure S2. |

Human data:

| **Variable Name** | **Value Explanation** |
| --- | --- |
| Play_condition | ‘0’=normal; ‘1’=reverse |
| Temporal_distance | 1 to 25 correspond to 25 TD levels |
| boundary | ‘0’=within; ‘1’=across |
| response_correct | ‘1’=correct; ‘0’=incorrect |
| mouse.time | RT |
| delay1 | The delay time of correct answer (ms) |
| delay2 | The delay time of the other frame (ms) |
| Fresh/Repeat | 1 to 5 correspond to 5 exposure levels. |
| Nonprimate/Primate | ‘0’=primate; ‘1’=non-primate |
| Monkey | 1 to 7 correspond to different human subjects. |
| chosen_frame_location | The frame number of which the monkey has chosen in each trial. |
