# Supplementary material for: Behavioral evidence for memory replay of video episodes in the macaque
Source: eLife. 2020 Apr 20;9:e54519. doi: 10.7554/eLife.54519 (PMC7234809; doi:10.7554/eLife.54519)
Supplement: Supplementary file 2. — The three panels correspond to analyses performed using all trials (top), only correct trials (middle), and only incorrect trials (bottom). The same slope patterns were observed irrespective of correctness, as is consistent with the analyses of slopes of reciprocal latency as a function of chosen frame location for each monkey. Related to Figure 2—figure supplement 1A. [file elife-54519-supp2.docx]

| Monkeys | Beta | SEM | t-statistics | *p*-value |  | 95% confidence interval  Lower Upper | |
| --- | --- | --- | --- | --- | --- | --- | --- |
| Slope of reciprocal latency/temporal similarity tested against zero (all trials) | | | | | | | |
| Jupiter | 0.257 | 0.026 | 9.874 | <0.001 |  | 0.206 | 0.308 |
| Mars | 0.434 | 0.029 | 15.200 | <0.001 |  | 0.378 | 0.490 |
| Saturn | 0.202 | 0.031 | 6.485 | <0.001 |  | 0.141 | 0.263 |
| Mercury | 0.239 | 0.059 | 4.019 | <0.001 |  | 0.122 | 0.355 |
| Uranus | 0.191 | 0.026 | 7.263 | <0.001 |  | 0.139 | 0.243 |
| Neptune | 0.245 | 0.037 | 6.675 | <0.001 |  | 0.173 | 0.317 |
| Slope of reciprocal latency/temporal similarity tested against zero (correct trials) | | | | | | | |
| Jupiter | 0.270 | 0.037 | 7.358 | <0.001 |  | 0.198 | 0.342 |
| Mars | 0.392 | 0.040 | 9.806 | <0.001 |  | 0.314 | 0.471 |
| Saturn | 0.145 | 0.043 | 3.364 | <0.001 |  | 0.061 | 0.230 |
| Mercury | 0.371 | 0.078 | 4.756 | <0.001 |  | 0.218 | 0.525 |
| Uranus | 0.204 | 0.036 | 5.641 | <0.001 |  | 0.133 | 0.275 |
| Neptune | 0.187 | 0.051 | 3.653 | <0.001 |  | 0.087 | 0.288 |
| Slope of reciprocal latency/temporal similarity tested against zero (Incorrect trials) | | | | | | | |
| Jupiter | 0.246 | 0.037 | 6.629 | <0.001 |  | 0.173 | 0.318 |
| Mars | 0.481 | 0.041 | 11.771 | <0.001 |  | 0.400 | 0.561 |
| Saturn | 0.257 | 0.045 | 5.710 | 0.002 |  | 0.169 | 0.345 |
| Mercury | 0.061 | 0.089 | 0.689 | 0.491 |  | -0.113 | 0.235 |
| Uranus | 0.167 | 0.038 | 4.447 | <0.001 |  | 0.093 | 0.241 |
| Neptune | 0.301 | 0.053 | 5.704 | <0.001 |  | 0.198 | 0.405 |
|  |  |  |  |  |  |  |  |
|  |  |  |  |  |  |  |  |
