# Supplementary material for: Behavioral evidence for memory replay of video episodes in the macaque
Source: eLife. 2020 Apr 20;9:e54519. doi: 10.7554/eLife.54519 (PMC7234809; doi:10.7554/eLife.54519)
Supplement: Supplementary file 3. [file elife-54519-supp3.docx]

| Monkey | Model | d.f. | *F* | *p*-value |  | Human | Model | d.f. | *F* | *p*-value |
| --- | --- | --- | --- | --- | --- | --- | --- | --- | --- | --- |
| Jupiter | CFL | (1,255) | 25.43 | <0.001 |  | Subject 1 | CFL | (1,214) | 3.58 | 0.060 |
|  | CFL+CFL^2^ | (2,254) | 14.88 | <0.001 |  |  | CFL+CFL^2^ | (2,213) | 2.28 | 0.105 |
|  | CFL+ CFL^2^ +CFL^3^ | (3,253) | 12.20 | <0.001 |  |  | CFL+ CFL^2^ +CFL^3^ | (3,212) | 4.06 | 0.008 |
| Mars | CFL | (1,252) | 76.49 | <0.001 |  | Subject 2 | CFL | (1,224) | 1.12 | 0.292 |
|  | CFL+CFL^2^ | (2,251) | 38.57 | <0.001 |  |  | CFL+CFL^2^ | (2,223) | 1.57 | 0.211 |
|  | CFL+ CFL^2^ +CFL^3^ | (3,250) | 42.76 | <0.001 |  |  | CFL+ CFL^2^ +CFL^3^ | (3,222) | 7.87 | <0.001 |
| Saturn | CFL | (1,261) | 13.25 | <0.001 |  | Subject 3 | CFL | (1,214) | 0.62 | 0.431 |
|  | CFL+CFL^2^ | (2,260) | 6.60 | 0.002 |  |  | CFL+CFL^2^ | (2,213) | 9.36 | <0.001 |
|  | CFL+ CFL^2^ +CFL^3^ | (3,259) | 4.88 | 0.003 |  |  | CFL+ CFL^2^ +CFL^3^ | (3,212) | 9.92 | <0.001 |
| Mercury | CFL | (1,255) | 34.88 | <0.001 |  | Subject 4 | CFL | (1,202) | 0.56 | 0.454 |
|  | CFL+CFL^2^ | (2,254) | 17.90 | <0.001 |  |  | CFL+CFL^2^ | (2,201) | 0.28 | 0.755 |
|  | CFL+ CFL^2^ +CFL^3^ | (3,253) | 13.46 | <0.001 |  |  | CFL+ CFL^2^ +CFL^3^ | (3,200) | 3.52 | 0.016 |
| Uranus | CFL | (1,258) | 19.69 | <0.001 |  | Subject 5 | CFL | (1,226) | 0.20 | 0.657 |
|  | CFL+CFL^2^ | (2,257) | 9.82 | <0.001 |  |  | CFL+CFL^2^ | (2,225) | 0.36 | 0.697 |
|  | CFL+ CFL^2^ +CFL^3^ | (3,256) | 6.57 | <0.001 |  |  | CFL+ CFL^2^ +CFL^3^ | (3,224) | 3.65 | 0.013 |
| Neptune | CFL | (1,256) | 10.25 | 0.002 |  | Subject 6 | CFL | (1,218) | 0.61 | 0.436 |
|  | CFL+CFL^2^ | (2,255) | 5.38 | 0.005 |  |  | CFL+CFL^2^ | (2,217) | 0.65 | 0.522 |
|  | CFL+ CFL^2^ +CFL^3^ | (3,254) | 4.34 | 0.005 |  |  | CFL+ CFL^2^ +CFL^3^ | (3,216) | 4.46 | 0.005 |
|  |  |  |  |  |  | Subject 7 | CFL | (1,208) | 1.78 | 0.184 |
|  |  |  |  |  |  |  | CFL+CFL^2^ | (2,207) | 2.79 | 0.063 |
|  |  |  |  |  |  |  | CFL+ CFL^2^ +CFL^3^ | (3,206) | 3.94 | 0.009 |

CFL, chosen frame location, d.f., Degrees of freedom.
