# Supplementary material for: Behavioral evidence for memory replay of video episodes in the macaque
Source: eLife. 2020 Apr 20;9:e54519. doi: 10.7554/eLife.54519 (PMC7234809; doi:10.7554/eLife.54519)
Supplement: Supplementary file 4. — Related to Figure 2—figure supplement 1B. [file elife-54519-supp4.docx]

| Subjects | Beta | SEM | t-statistics | *p*-value |  | 95% confidence interval  Lower Upper | |
| --- | --- | --- | --- | --- | --- | --- | --- |
| Slope of reciprocal latency/temporal similarity tested against zero | | | | | | | |
| Subject 1 | -0.120 | 0.026 | -4.609 | <0.001 |  | -0.171 | -0.069 |
| Subject 2 | -0.100 | 0.016 | -6.249 | <0.001 |  | -0.131 | -0.068 |
| Subject 3 | -0.169 | 0.026 | -6.410 | <0.001 |  | -0.221 | -0.118 |
| Subject 4 | -0.110 | 0.020 | -5.629 | <0.001 |  | -0.148 | -0.072 |
| Subject 5 | -0.102 | 0.024 | -4.319 | <0.001 |  | -0.149 | -0.056 |
| Subject 6 | -0.044 | 0.023 | -1.919 | 0.055 |  | -0.089 | 0.001 |
| Subject 7 | -0.133 | 0.026 | -5.105 | <0.001 |  | -0.184 | -0.082 |
| Slope of reciprocal latency/chosen frame location tested against zero | | | | | | | |
| Subject 1 | 0.022 | 0.021 | 1.009 | 0.313 |  | -0.020 | 0.064 |
| Subject 2 | 0.025 | 0.013 | 2.003 | 0.045 |  | 0.001 | 0.050 |
| Subject 3 | 0.100 | 0.022 | 4.517 | <0.001 |  | 0.057 | 0.144 |
| Subject 4 | 0.076 | 0.016 | 4.809 | <0.001 |  | 0.045 | 0.106 |
| Subject 5 | 0.016 | 0.019 | 0.865 | 0.387 |  | -0.021 | 0.054 |
| Subject 6 | -0.004 | 0.019 | -0.217 | 0.828 |  | -0.041 | 0.033 |
| Subject 7 | 0.053 | 0.021 | 2.505 | 0.012 |  | 0.011 | 0.094 |
|  |  |  |  |  |  |  |  |
