# Supplementary material for: Behavioral evidence for memory replay of video episodes in the macaque
Source: eLife. 2020 Apr 20;9:e54519. doi: 10.7554/eLife.54519 (PMC7234809; doi:10.7554/eLife.54519)
Supplement: Supplementary file 5. — For ease of comparison, we computed the respective ΔBIC to index the strength of evidence for each model. Note that the model with the lowest BIC is the winning model. In all 6 monkeys, the shift model is superior to the other three models, whereas this effect is not consistent in the humans. Related to Figure 5. [file elife-54519-supp5.docx]

| Condition | | BIC | | | | delta_BIC | | | |
| --- | --- | --- | --- | --- | --- | --- | --- | --- | --- |
|  |  | null | two fits | swivel | shift | null | two fits | swivel | shift |
|  | Jupiter | -67157.39 | -67603.99 | -67543.25 | -67615.23 | 457.83 | 11.24 | 71.98 | 0 |
|  | Mars | -63948.06 | -65937.89 | -65769.84 | -65948.72 | 2000.65 | 10.82 | 178.88 | 0 |
|  | Saturn | -61307.14 | -61929.72 | -61853.77 | -61938.70 | 631.56 | 8.98 | 84.93 | 0 |
|  | Mercury | -63904.96 | -64047.31 | -64042.94 | -64057.52 | 152.56 | 10.20 | 14.57 | 0 |
|  | Uranus | -67652.66 | -67850.84 | -67841.34 | -67864.67 | 212.01 | 13.83 | 23.34 | 0 |
|  | Neptune | -56695.53 | -58327.76 | -58037.15 | -58337.23 | 1641.70 | 9.47 | 300.07 | 0 |
| Across VS Within | Subject 1 | -29720.89 | -29209.81 | -29649.71 | -29720.89 | 3358.60 | 0 | 511.08 | 71.18 |
|  | Subject 2 | -30537.49 | -30516.16 | -30520.98 | -30537.49 | 2243.88 | 0 | 21.33 | 16.51 |
|  | Subject 3 | -28270.09 | -28133.70 | -28274.72 | -28274.72 | 2970.60 | 4.63 | 141.02 | 0 |
|  | Subject 4 | -30129.85 | -29632.63 | -30116.62 | -30129.85 | 6115.33 | 0 | 497.22 | 13.22 |
|  | Subject 5 | -29043.03 | -29039.37 | -28995.64 | -29043.03 | 1968.10 | 0 | 3.67 | 47.39 |
|  | Subject 6 | -28973.84 | -28940.92 | -28828.03 | -28973.84 | 3265.97 | 0 | 32.92 | 145.81 |
|  | Subject 7 | -28999.94 | -28741.54 | -29012.43 | -29012.43 | 5136.62 | 12.50 | 270.89 | 0 |
